# Impact of acute kidney injury on coagulation in adult minimal change nephropathy

**DOI:** 10.1097/MD.0000000000005366

**Published:** 2016-11-18

**Authors:** Meng-Jie Huang, Ri-bao Wei, Ting-yu Su, Yang Wang, Qing-ping Li, Xi Yang, Xiao-meng Lv, Xiang-mei Chen

**Affiliations:** Department of Nephrology, Chinese PLA General Hospital, Chinese PLA Institute of Nephrology, State Key Laboratory of Kidney Diseases, National Clinical Research Center for Kidney Diseases, Beijing, China.

**Keywords:** acute kidney injury, coagulation, minimal change disease, nephrotic syndrome, thromboembolic events

## Abstract

A hypercoagulable state exists in patients with nephrotic syndrome (NS), which more easily leads to venous thromboembolism (VTE). However, whether acute kidney injury (AKI), a common complication of NS, affects the hypercoagulable state and VTE has rarely been elucidated. In this study, we aimed to explore coagulation changes and analyze relevant influencing factors in NS-AKI patients.

A total of 269 consecutive NS patients with minimal change disease (MCD) between 2011 and 2016 were included in this observational study. Ninety-one cases were in the AKI group and 178 cases in the non-AKI group. The 1:1 propensity score matching (PSM) method was applied to match the baseline information. The coagulation biomarkers were compared, and the thrombosis events were recorded. Linear correlation was performed to detect any relation between D-dimer and clinical data.

The PSM method gave matched pairs of 88 MCD patients with AKI and non-AKI patients, resulting in no differences in baseline information. The D-dimer, fibrinogen, and thromboelastography parameters maximum amplitude (MA), G values of the MCD-AKI patients were significantly higher than the levels of the MCD patients without AKI (D-dimer: 1.8 [1.0, 3.3] vs 1.1 [0.6, 1.7] mg/L, *P* < 0.001; fibrinogen: 7.0±2.0 vs 6.5 ± 1.4 g/L, *P* = 0.036; MA: 74.6 ± 5.0 vs 70.5 ± 5.3 mm, *P* = 0.020; G: 15.7 ± 5.3 vs 12.5 ± 3.3, *P* = 0.034). For the MCD patients, the serum creatinine, white blood cell count, and interleukin-6 levels in the patients with D-dimers >1 mg/L were significantly higher than those of patients with D-dimers ≤1 mg/L. The correlation analysis showed that the D-dimer level was correlated with serum creatinine, white blood cell count, and interleukin-6 (*r* = 0.410, *P* =  < 0.001; *r* = 0.248, *P* =  < 0.001; *r* = 0.306, *P* =  < 0.001, respectively). Five deep vein thrombosis events occurred in the AKI group and 1 pulmonary embolism event occurred in the non-AKI group after adjusting the propensity score value. AKI appeared to have an association with higher incidence of VTE, but the difference was not statistically significant (RR: 4.9, 95% CI: 0.6–42.7, *P* = 0.154).

The MCD-NS patients complicated with AKI had a more severe hypercoagulable state, which might be associated with the active inflammation of AKI that mediated activation of the coagulation system.

## 1 Introduction

Nephrotic syndrome (NS) is characterized by massive proteinuria (≥3.5 g/day), hypoalbuminemia (<30 g/L), edema, and hyperlipidemia.^[1]^ Minimal change disease (MCD), a kind of glomerulonephritis, is one of the most common causes of NS due to the glomerular filtration barrier damage and heavy proteinuria consequent. A hypercoagulable state exists in MCD patients, which easily leads to venous thromboembolism (VTE) (manifesting as deep vein thrombosis or pulmonary embolism) that carry significant morbidity and mortality.

Patients with MCD often develop acute kidney injury (AKI) which is characterized by a rapid decline in renal function and retention of nitrogenous waste products.^[2,3]^ Renal dysfunction easily leads to coagulation disorders due to the retention of metabolic toxins and an inflammatory status.^[4]^ However, whether AKI affects the hypercoagulable state and thromboembolism in MCD patients complicated with AKI is unclear.

In previous studies, Waldman et al^[5]^ and Chen et al^[6]^ showed that MCD patients with AKI were more likely to be older and have a lower serum albumin level. Age and the albumin level were the confounding factors that affect coagulation and thromboembolism.^[7–9]^ Thus, we introduced the propensity score matching (PSM) method which has been widely used in the observational studies to adjust for differences in the baseline patient characteristics between the study and control groups and to reduce the biases and potential confounding factors.^[10]^

This study applied the PSM method to investigate changes in the coagulation function in MCD-NS patients with AKI, record thromboembolism, and analyze the relevant factors that influence coagulation.

## 2 Study design and patient selection

### 2.1 Study objects

This study was a retrospective observational cohort and complies with STROBE recommendations. Consecutive cases of adult NS patients with the MCD pathological type diagnosed by renal biopsy at the Chinese PLA General Hospital from January 2011 to June 2016 were included. The exclusion criteria were as follows: (1) <18 years of age; (2) received hormone or immunosuppressive therapy prior to admission; (3) complicated with a malignant tumor, acute liver injury, pregnancy or history of thromboembolism or hemorrhage disorder; (4) received anticoagulant therapy (i.e., heparin/warfarin) 1 week before the AKI occurred; and (5) a history of chronic kidney disease. Additionally, AKI cases due to the application of nonsteroidal anti-inflammatory drugs, antibiotics, contrast agents or other potentially nephrotoxic drugs, the presence of renal vein thrombosis, and rental hypoperfusion caused by a low blood volume and infection were excluded.^[11,12]^ All renal-biopsy-patients had signed the research protocol of Renal Clinical Database Establishment when hospitalized, allowing their data for clinical research and the research was approved by the ethics committee of the General Hospital of the Chinese People's Liberation Army.

### 2.2 AKI diagnosis and classification

The cases were diagnosed and classified according to the *2012 Kidney Disease: Improving Global Outcomes (KDIGO) clinical practice guideline for acute kidney injury*.^[13]^ As this study was a retrospective study, the urine changes in the patients during the AKI period were not available. Therefore, AKI was defined and classified based on the increase in serum creatinine. The specific grading standard is shown in Table 1.

**Table 1:**
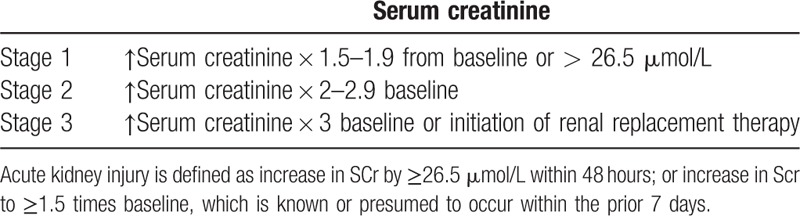
Definition and classification of acute kidney injury.

### 2.3 Data collection

Clinical and laboratory data were collected from the nearest time points to the patients’ peak serum creatinine values prior to treatment with glucocorticoids and immunosuppressants and renal replacement therapy.

### 2.4 General information

The age, gender, hemoglobin, white blood cell count, neutrophil ratio (N%), platelet count, serum albumin, serum creatinine, total serum cholesterol, triglycerides, erythrocyte sedimentation rate (ESR), and interleukin-6 of the MCD patients were recorded. The estimated glomerular filtration rate (eGFR) was calculated based on the EPI (epidemiology collaboration) equation.^[14]^

### 2.5 Conventional coagulation test

The coagulation index included the following: (1) plasma prothrombin time (PT) (the normal range of PT is between 11 and 15 seconds), (2) activated partial thromboplastin time (APTT) (the normal range of APTT is between 30 and 45 seconds), (3) D-dimer (the normal range of D-dimer is between 0 and 0.5 mg/L), (4) fibrinogen (the normal range of FIB is between 2 and 4 g/L), (5) antithrombin 3 (the normal range of antithrombin 3 is between 80 and 120%). The APTT, PT, and fibrinogen were analyzed by the magnetic bead assay. Antithrombin III was analyzed by the chromogenic substrate assay. The D-dimer content was measured by immunoturbidimetry using a device and reagents purchased from Stago (France). Since an elevated D-Dimer was not only present in VTE, but also in stroke, disseminated intravascular coagulation, pregnancy, liver disease, lipid disorders, heart disease and so on, we excluded the NS-AKI patients with the mentioned comorbidities.

### 2.6 Thromboelastography

Interpretation of thromboelastography data was based on a global assessment of coagulation incorporating the cumulative effect of the interactions at various levels between plasma components (clotting proteins) and cellular components (platelets, red and white blood cells, and microparticles) of coagulation, thus allowing a dynamic assessment at different stages of clot formation/fibrin polymerization (clot initiation, amplification, and propagation to fibrinolysis). Parameters included the following: (1) reaction time (R)—time from the start of the test to a thromboelastography amplitude of 2 mm, reflecting the combined effect of coagulation factors involved in the initiation of hemostasis; (2) K-time (K)—the period from the thromboelastography amplitude of 2 mm to the curve reaches amplitude of 20 mm, which measures the rate of clot formation (fibrin cross-linking); (3) maximum amplitude (MA)—indicative of the strength of clot that reflects the cross interaction between platelet functions and coagulation; (4) G—the representation of overall clot elasticity. G was calculated in formulae: *G* = (5000 × MA)/(100–MA). A clot with high elasticity was more resistant to both mechanical and enzymatic degradation.^[15,16]^ The hypercoagulable state was the following: shortened R time, increased K, MA, and G.

### 2.7 Venous thromboembolism

The incidence of VTE in the MCD patients was recorded before the complete remission of NS. Evidence suggested that patients with suspected VTE should be managed with a diagnostic strategy that includes clinical pretest probability in the form of prediction scores, D-dimer test, and appropriate clinical imaging results.^[17]^ Since this was a retrospective study, the individual clinical imaging method used for thromboembolism detection was determined by the clinician.

### 2.8 Statistical methods

Continuous variables were expressed as mean ± standard deviation or median with interquartile range. The independent sample *t* test or Mann–Whitney test was used for continuous variables and the chi-squared test for categorical variables. 95% confidence intervals of the group difference were calculated. The PSM process was achieved using the PSM extension program in SPSS with the occurrence of AKI as the dependent variable and age, serum albumin as the independent variables. The propensity scores were estimated by logistic regression. Matching was performed using the method of 1:1 nearest neighbor matching, and the excellence of the matching results in the process was assured by defining the caliper values. The standard differences of the covariates between the 2 groups were set to 0.1. Linear correlation was used to detect any relation between D-dimer and clinical data. All data were statistically analyzed using the SPSS software, version 19.0 (Chicago, IL), and *P* values <0.05 were considered statistically significant.

## 3 Results

### 3.1 AKI incidence in the MCD patients

**Figure 1:**
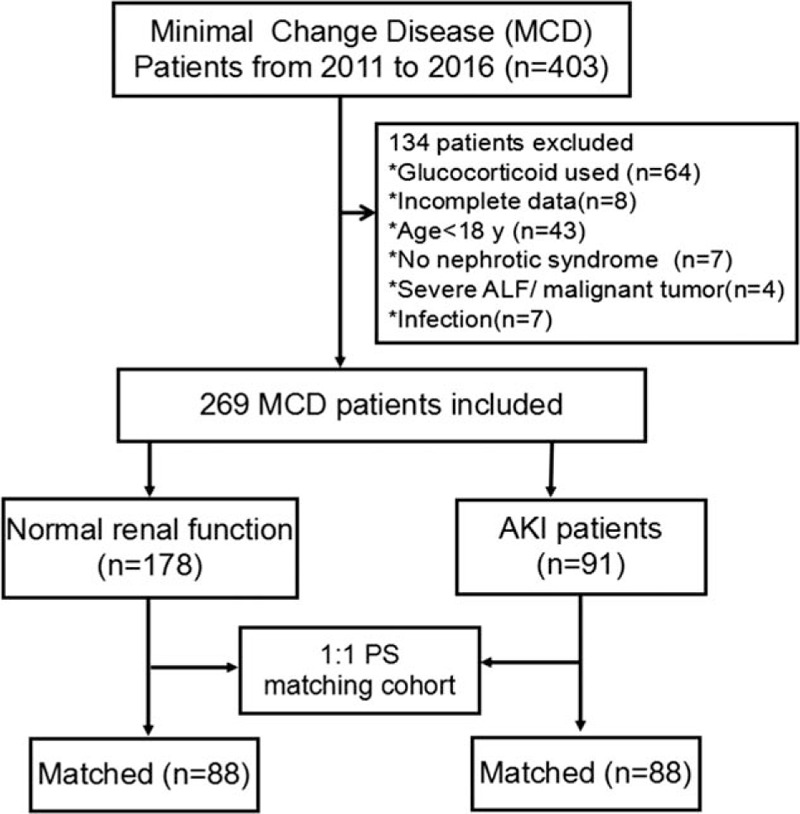
Derivation and flow diagram of the minimal change disease cohort.

From January 2011 to June 2016, the Department of Nephrology, Chinese PLA General Hospital diagnosed 403 patients with MCD by renal biopsy, of whom 269 patients had complete clinical data and met the inclusion and exclusion criteria to enroll in this study (Fig. 1). A total of 91 out of the 269 MCD patients showed AKI (34%), including 30 cases of stage 1 AKI, 32 cases of stage 2 AKI, and 29 cases of stage 3 AKI.

### 3.2 Characteristics of the MCD-AKI and MCD-non AKI patients

The baseline characteristics of the patients were shown in Table 2. In the unmatched cohort, the MCD patients with AKI were significantly older (95% confidence interval [CI] of the difference: 4.7–12.7, *P* < 0.001) and had lower serum albumin (95% CI: –2.1 to –0.1, *P* = 0.025) than the non-AKI patients. The baseline hemoglobin, platelets, cholesterol, and triglycerides did not differ between groups. The 1:1 PSM yielded 88 pairs of matched patients with no differences in age and serum albumin. However, the proportion of males in the AKI-MCD and the white blood cell, neutrophil (%), and interleukin-6 levels of the patients before and after matching were significantly higher than those of the patients in the non-AKI group (95% CI: 0.7–1.9, *P* < 0.001; 95% CI: 4.4–11.3, *P* < 0.001; 95% CI: 1.6–12.4, *P* < 0.001,respectively).

**Table 2:**
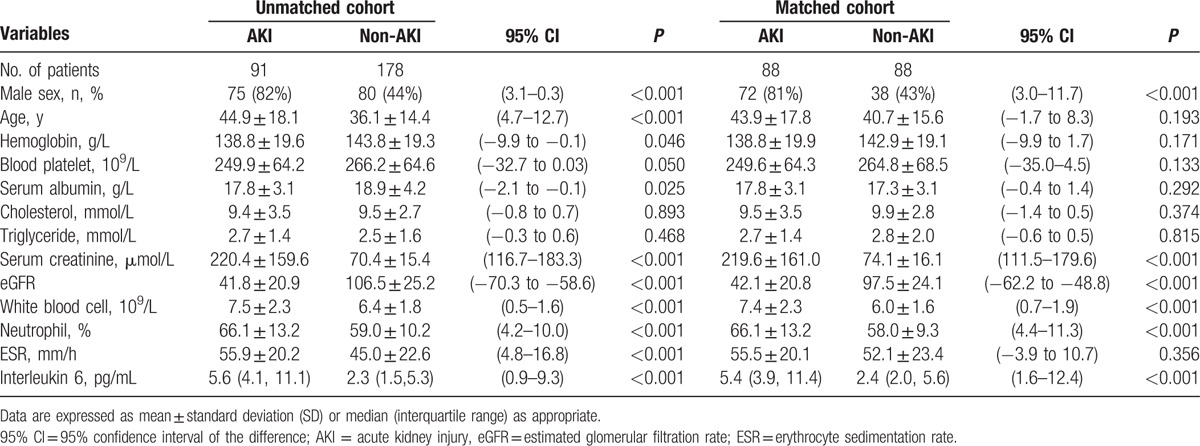
Baseline characteristics (before and after propensity score 1:1 matching).

### 3.3 Conventional indicators of coagulation

Table 3 showed the comparison of the indicators of coagulation between the non-AKI and AKI-MCD patients. The results showed that the D-dimer, which was a useful aid for the diagnosis of thromboembolism, and fibrinogen levels were significantly higher in the MCD-AKI patients than in the non-AKI patients (95% CI: 0.1–1.0, *P* = 0.036; 95% CI: 0.8–2.4, *P* < 0.001,respectively), whereas APTT, PT, and antithrombin 3 were not significantly different between the 2 groups.

**Table 3:**
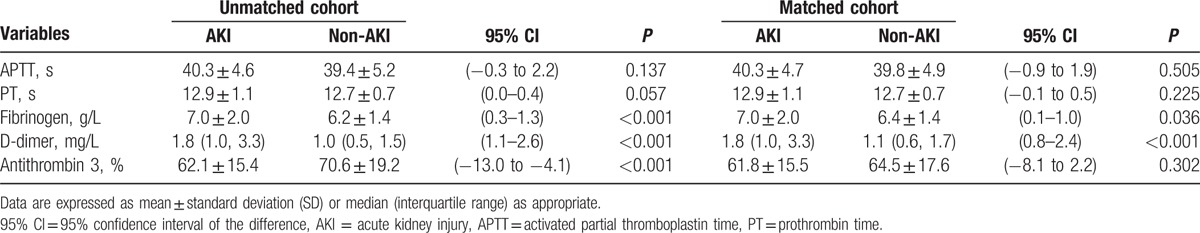
Coagulation index profiles before and after propensity score 1:1 matching.

### 3.4 Thromboelastography

**Figure 2:**
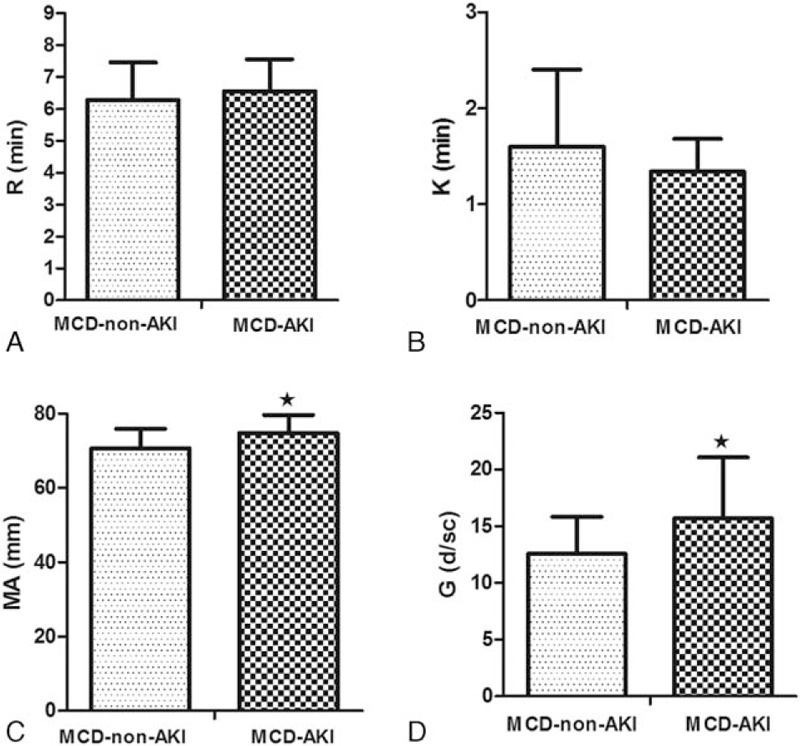
TEG parameters of MCD-non-AKI vs MCD-AKI. ∗*P* < 0.05, vs MCD-non-AKI. (A) *R* value, (B) *K* value, (C) MA value, (D) *G* value. AKI = acute kidney injury, MA = maximum amplitude, MCD = minimal change disease, *R* = reaction time, TEG = thromboelastography.

The thromboelastography parameters from the MCD patients with and without AKI were analyzed after matching (Fig. 2). The maximum amplitude (MA) and *G* values of the patients in the MCD-AKI group were higher than those of the MCD patients with normal renal functions (MA: 74.6 ± 5.0 vs 70.5 ± 5.3 mm, *P* = 0.020; G: 15.7 ± 5.3 vs 12.5 ± 3.3, *P* = 0.034). The *R* value and *K* value were not significantly different between the groups.

### 3.5 Associations between the coagulation index and clinical features

Previous studies used a D-dimer >1 mg/L as an indication of anticoagulation in NS patients.^[18]^ Therefore, we compared the clinical indicators of a D-dimer >1 mg/L and ≤1 mg/L for the MCD-NS patients. Table 4 showed that the serum creatinine and related inflammatory indicators (white blood cell and interleukin-6) levels were significantly higher in the patients in the D-dimer >1 mg/L group than in the patients in the D-dimer ≤1 mg/L group (95% CI: 35.6–99.3, *P* < 0.001; 95% CI: 0.1–1.3, *P* = 0.047; 95% CI: 0.3–10.9, *P* = 0.039, respectively) (Table 4).

**Table 4:**
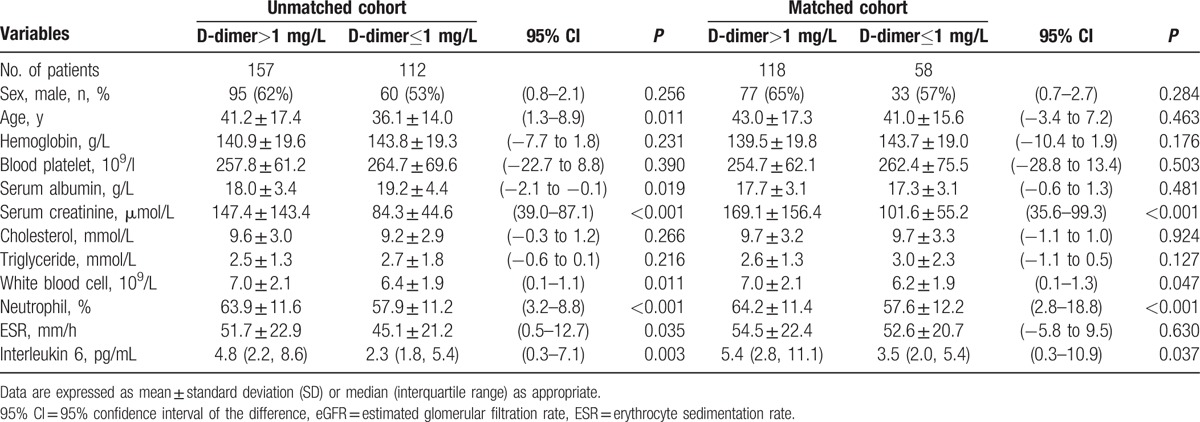
Comparison of clinical features of minimal change disease patients with D-dimer>1 or ≤1 mg/L.

Additionally, the correlation analysis showed that the D-dimer level of the MCD-NS patients was positively correlated with the AKI stage and the serum creatinine, white blood cell, and interleukin-6 levels (*r* = 0.434, *P* =  < 0.001; *r* = 0.430, *P* =  < 0.001; *r* = 0.209, *P* = <0.001; and *r* = 0.340, *P* = <0.001, respectively). Even after adjusting the PS value, the D-dimer level was still positively correlated with the AKI stage and the serum creatinine, white blood cell, and interleukin-6 levels (*r* = 0.395, *P* = <0.001; *r* = 0.410, *P* = <0.001; *r* = 0.248, *P* = <0.001; and *r* = 0.306, *P* = <0.001, respectively).

### 3.6 Thromboembolic events

The MCD patients were followed-up, and VTE that occurred prior to complete NS remission were recorded. Among the 269 MCD patients, follow-up records were available for 208 cases (77 in the AKI group and 161 in the non-AKI group), with a follow-up rate of 77%. The average remission time of the MCD patients with and without AKI were 51 (33, 68) and 40 (23, 60) days, respectively. The follow-up results showed that deep vein thrombosis in the lower limbs occurred in 6 patients with AKI prior to NS remission (4 cases of stage 3 AKI, 1 case of stage 2 AKI, and 1 case of stage 1 AKI), whereas a pulmonary embolism event occurred in 1 MCD patient with non-AKI. The risk ratio (RR) of VTE in unmatched cohort was 10.9 (95% CI: 1.3–93.0, *P* = 0.028). However, there remained 5 events in the MCD-AKI group (n = 73) and 1 event in the MCD-Non-AKI group (n = 64) after adjusting the propensity score value. AKI appeared to have an association with higher incidence of VTE, but the difference was not statistically significant (RR: 4.9, 95% CI: 0.6–42.7, *P* = 0.154) (Table 5).

**Table 5:**
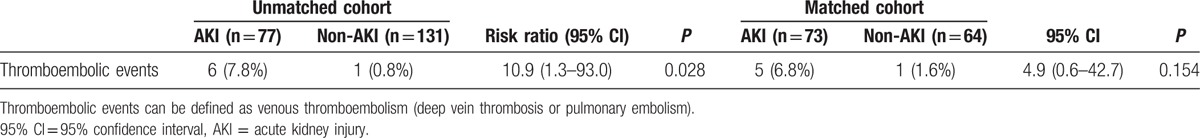
Risk ratio for thromboembolic events in patients who were available for follow-up.

## 4 Discussion

A hypercoagulable state exists in patients with NS, which more easily leads to thromboembolic events. However, whether AKI affects the hypercoagulable state and thromboembolic events in NS patients has rarely been reported. The present study explored for the first time the changes in coagulation in NS patients with AKI, recorded thrombosis events, and analyzed the relevant influencing factors.

The study found that the D-dimer, fibrinogen, thromboelastography parameters and the incidence of VTE prior to complete NS remission of the MCD patients with AKI were significantly higher than those of the non-AKI patients, suggesting that the MCD-NS patients with AKI had more obvious hypercoagulable state. As uremic toxins could reduce platelet and vessel wall functions, patients with renal failure were often considered to have a high risk of hemorrhage.^[19–21]^ However, due to the rapid progress of AKI, it was difficult for the toxins to fully accumulate in a short time. Thus, metabolic toxin was not the main factor affecting the change of coagulation in AKI patients.^[22,23]^

AKI was associated with a systemic inflammatory syndrome.^[24]^ This study found that the Scr, WBC, and IL-6 levels of the MCD patients with D-dimers >1 mg/L were significantly higher than the levels of the patients with D-dimers ≤1 mg/L. The correlation analysis showed that the D-dimer level was positively correlated with the white blood cell, interleukin-6, and ESR inflammatory indicators. Therefore, we hypothesized that the hyperactive coagulation functions of the patients with MCD-AKI might be related to systemic inflammation. Extensive cross-linking existed between the coagulation system and the immune and inflammatory system.^[25]^ The inflammatory response could stimulate the generation of thrombin mediated by tissue factors, reduce anticoagulants, and inhibit fibrinolysis, thereby activating the coagulation system and causing an increased risk of thrombosis.^[26,27]^

However, the routine detection of the coagulation indicators in the present study showed that APTT and PT were not significantly different in MCD patients with and without AKI. In fact, the homeostasis of coagulation was mutually balanced and adjusted by multiple factors, including blood vessels, platelets, the coagulation system, the anticoagulation system, and the fibrinolysis system, whereas APTT and PT only reflected the coagulation function and were not suitable for the evaluation of the real state of coagulation. Thromboelastography was a detection method that reflects the entire process of blood coagulation. When the data from patients with thromboelastography testing were analyzed, the results showed that the MA value and *G* value of the MCD patients with AKI were higher than those of the MCD patients with non-AKI, suggesting that the coagulation of the MCD-AKI patients was more hyperactive.

This study explored for the first time the coagulation and VTE of NS-AKI patients with the MCD pathological type. The study had some limitations. (1) Because this study was a retrospective study, the routine tests for many blood coagulation-related indicators (i.e., vascular endothelium, platelet function, coagulation factor levels, and anticoagulation system) and the imaging studies for thrombosis were not available, resulting in shortcomings in elucidating the entire coagulation process of MCD-AKI patients. Thus, the incidence of VTE might be underestimated. Further prospective research was still required to investigate the whole hemostatic changes in MCD-AKI. (2) Although the PSM method was used to balance the major confounding variables, many unknown confounding variables might still affect the final results.

This study suggested that MCD-NS patients with AKI had a more severe hypercoagulable state, which might be related to the active immune inflammation of AKI that mediates activation of the coagulation system. And the findings might guide clinicians toward more effective antithrombotic therapy.
